# The geographic embedding of online echo chambers: Evidence from the Brexit campaign

**DOI:** 10.1371/journal.pone.0206841

**Published:** 2018-11-02

**Authors:** Marco Bastos, Dan Mercea, Andrea Baronchelli

**Affiliations:** 1 Department of Sociology, City, University of London, London, United Kingdom; 2 Department of Mathematics, City, University of London, London, United Kingdom; University of Warwick, UNITED KINGDOM

## Abstract

This study explores the geographic dependencies of echo-chamber communication on Twitter during the Brexit campaign. We review the evidence positing that online interactions lead to filter bubbles to test whether echo chambers are restricted to online patterns of interaction or are associated with physical, in-person interaction. We identify the location of users, estimate their partisan affiliation, and finally calculate the distance between sender and receiver of @-mentions and retweets. We show that polarized online echo-chambers map onto geographically situated social networks. More specifically, our results reveal that echo chambers in the Leave campaign are associated with geographic proximity and that the reverse relationship holds true for the Remain campaign. The study concludes with a discussion of primary and secondary effects arising from the interaction between existing physical ties and online interactions and argues that the collapsing of distances brought by internet technologies may foreground the role of geography within one’s social network.

## Introduction

Literature on online social networks defines echo chambers as a process of self-selection that confines communication to ideologically-aligned cliques [1, 2]. Political communication studies have advanced research on echo-chambers which are seen as problematic for democracy as they engender political polarization [3], reinforce individual views and preferences [4], and foreclose deliberation [5]. Evidence shows that filter bubbles and echo chambers affect a limited subset of the online population [6, 7] and that politically homogeneous echo chambers are embedded in processes of political polarization and selective exposure leading to negative attitudes about out-group members [8, 9].

The prevailing narrative about politically homogeneous echo chambers argues that the interaction patterns existing in social platforms lead users to engage with political content resonating with them [3]. The ideological clustering observed in politically homogeneous echo chambers and algorithmic filter bubbles thus stands in contrast to the diversity of opinions found in face-to-face interactions. It ultimately jeopardizes political compromise as ill-informed individuals inhabit different networks in a society increasingly segregated along polarized partisan lines [10]. Transposed to the network of tweets about the U.K. E.U. membership referendum, we would expect to find echo chambers as a communication artifact resulting from online discussion alone. Conversely, we would not expect the geographic locations of users to play a significant role in the formation of echo chambers, as echo chambers result from social media interactions which are unencumbered by geographic space.

This paper explores whether echo chambers depart from phenomena occurring in offline networks clustering. The null hypothesis is that the forces underpinning echo chambers are misrepresented: instead of resulting from interaction in social platforms, echo chambers would reproduce the structural political polarization found in physical social networks. Our hypothesis is informed by evidence that bidirectional association between geography and network formation is a significant driver of tie-selection and retention [11]. Furthermore, geographic proximity affects tie-formation mechanisms associated both with opportunity and preferences, as physical places can be conceived of as a bundle of resources and opportunities with the additional characteristic of spatial contiguity [12].

In summary, the hypothesis about the geographic embedment of echo chambers draws from the homophily model which posits that individuals inhabiting physical communities are more likely to connect with others sharing similar social characteristics, so that cultural similarities and differences among people can be formalized as a function of geographic propinquity [11, 13]. Echo chambers moreover censor, disallow, or underrepresent competing views by enforcing social homogeneity much in line with the bandwagon effect predicted by the homophily model [14]. Finally, online social networks are more prone to homophily compared with offline networks, which are tied to physical locations where serendipitous exposure to social diversity is more likely to happen [15, 16]. These factors driving homophily in online and offline networks allow for testing whether users engaging in echo-chamber communication during the Brexit debate are clustered in geographically homogeneous subgraphs.

## Background

The Brexit referendum campaign was held at a time of reported polarization among the electorate on the cultural and economic costs and benefits of E.U. membership [17]. While older and culturally conservative voters protested the infringement on national sovereignty by European institutions, in addition to being concerned about the impact of E.U. workforce mobility on British society, a more liberal-minded electorate welcomed the collective decision-making at the heart of the European Union and accepted the inward E.U. migration into the U.K. as a source of greater diversity [18]. In the run-up to the referendum, the winning campaign to leave the Union foregrounded a culturally conservative message centered on the proposition to “take back control” from the E.U. by reasserting the sovereignty of the British Parliament and courts of justice and curtailing the free movement of E.U. labor. The vote to leave the E.U. further exposed a geographical splintering of the country. The metropolitan area of London, Scotland, and Northern Ireland voted Remain while the rest of England as well as Wales voted Leave [19].

Social anxieties surrounding echo chambers posit that social media is another force driving political polarization [20], with a growing body of observational evidence exploring the role of social media in stratifying users across information sources [21]. While the rapid growth of online social networks fostered an expectation of higher exposure to a variety of news and politically diverse information [22], they also increased the appetite for selective exposure in highly polarized social environments [23], with the sharing of controversial news items being particularly unlikely to take place in these contexts [24]. The filter bubble hypothesis encapsulates these claims by positing that social platforms deploy algorithms designed to quantify and monetize social interaction, narrowly confining it to a bubble algorithmically populated with information closely matching observed and expressed user preferences [25].

But researchers have also challenged the notion that social media cause selective exposure or ideological polarization, the latter being more pronounced in face-to-face interactions [26–28]. Exposure to diverse and even competing opinions on polarizing topics was found to occur on social media across various national contexts [7, 29, 30]. Similarly, social media was shown to be coextensive with more diverse personal networks which are more likely to include individuals from a different political party [31]. Even with scanty evidence linking filter bubbles and echo chambers to general social media communication, there is evidence of echo-chamber communication in several political contexts [4, 32–34]. In these settings, political information was more likely to be retweeted if received from ideologically similar sources [32] and cross-ideological information was unlikely to circulate in social clusters with a strong group identity [35], with clustering around partisan information sources being more prevalent among conservative than liberal voters [32, 36].

One possible explanation for the conflicting evidence on echo chambers is that politically homogeneous communication might reflect group formations inherited from offline social relations. These homophilic preferences can coexist with social media platforms that provide ideologically diverse networks [37]. As such, the boundaries of one’s network can be simultaneously permeated by echo chambers stemming from offline relationships while being exposed to competing opinions on polarizing topics that circulate on social media. Similar associational effects have been reported in the literature, with the relationship between spatial distance and users’ interaction on social media found to be significant, and friendship ties in densely connected groups arising at shorter spatial distances compared with social ties between members of different groups [38]. More importantly, research found social ties on Twitter to be constrained by geographical distance with an over-representation of ties confined to distances shorter than 100 kilometers [39]. These geographic constraints are likely to interact with the geographic patterning of the Brexit vote, which reveals spatial and associational segregation, with a spatial distribution in which people are more likely to talk to those who are categorically more similar to them.

Taking these considerations into account, we expect echo-chamber communication to be prevalent in the Brexit campaign (H1). In other words, we expect users identified with one side of the campaign to be more likely to engage with politically homogeneous groups. We refer to this pattern of echo chamber in which both sender and receiver are tweeting the same campaign as *in-bubble* interactions and we test this hypothesis for the Leave (H1a) and the Remain (H1b) campaigns. In view of the homophily dependencies reviewed hitherto, we also expect echo-chamber communication to be at least partially mirrored by geographic proximity. Therefore, we hypothesize that echo chambers are to be found predominantly in geographically proximate *in-bubbles* both in the Leave (H2a) and the Remain (H2b) campaigns. We refer to this geographically-bounded and politically homogeneous group as *neighboring in-bubble* communication in opposition to *cross-bubble*, when users interact with the other side of the campaign, and *out-bubble*, when users interact with a neutral user.

Thirdly, we take stock of the geographic factor possibly driving echo chambers and hypothesize that *in-bubbles* will cover shorter geographic distances compared with *out-* and *cross-bubbles*, both in the Leave (H3a) and Remain (H3b) campaigns. Hypotheses H2 and H3 are thus intrinsically connected: while the former explores whether echo chambers are more likely to span shorter distances, the latter (H3) probes whether distance is also associated with *cross-bubble* and *out-bubble* communication. Testing these hypotheses allows us to subsequently probe whether Leave interactions are more likely to be neighboring in-bubbles compared with Remain interactions (H4). Finally, and most importantly for the aims of this study, we postulated that echo-chamber communication (*in-bubble*) is associated with the geographic distance covered by messages in the Leave (H5a) and Remain (H5b) campaigns. As shown in Table 1, we proceed from a relatively simple hypothesis of whether echo-chamber communication occurred in the Brexit debate on Twitter towards the last hypothesis testing the geographic dependencies of echo chambers.

**Table 1:** Summary of tested hypotheses.

**H1**	H1a	Echo-chamber communication is prevalent in the Leave campaign
	H1b	Echo-chamber communication is prevalent in the Remain campaign
**H2**	H2a	Leave echo-chamber communication is marked by geographic proximity
	H2b	Remain echo-chambers are marked by geographic proximity
**H3**	H3a	Leave in-bubble communication covers shorter geographic distances
	H3b	Remain in-bubble communication covers shorter geographic distances
**H4**	H4	Leave interactions more likely to be neighboring in-bubbles
**H5**	H5a	Leave echo-chambers are associated with the geographic distance
	H5b	Remain echo-chambers are associated with the geographic distance

## Methods

We relied on the Twitter Streaming and REST Application Programming Interfaces (API), the endpoints Twitter offers to programmatically collect data, to amass a total of 5,099,180 tweets using a set of keywords and hashtags, including relatively neutral tags such as referendum, inorout, and euref, but more importantly, messages that used hashtags clearly aligned with the Leave campaign: voteleave, leaveeu, takecontrol, no2eu, betteroffout, voteout, britainout, beleave, iwantout, and loveeuropeleaveeu; and hashtags clearly aligned with the Remain campaign: strongerin, leadnotleave, votein, voteremain, moreincommon, yes2eu, yestoeu, betteroffin, ukineu, and lovenotleave. Reports of the most commonly used hashtags outside the search criteria were generated daily, inspected on a rolling basis, and added to the pool of terms whenever they proved to be relevant. This approach allowed for the expansion of the initial search criteria by consolidating the set of hashtags recurrently tweeted by users. The queried hashtags were parsed across multiple pools to avoid API filtering. Queries that exceeded the one-percent threshold were parsed across separate queries, cumulatively requiring a combination of twelve independent calls to the Streaming API. We subsequently removed messages tweeted before 15 April 2016, the starting date of the official campaign period, and after 24 June 2016, the end of the referendum campaign. The resulting dataset includes campaign-aligned hashtagged tweets that we leveraged to identify messages advocating each side of the referendum: the Vote Leave or Vote Remain campaigns.

Next, we identify the location of users in our dataset by triangulating information from geocoded tweets (subsequently reverse-geocoded), locations identified in their user profile (then geocoded), and information that appeared in their tweets. The triangulation prioritizes the signal with higher precision, hence geocoded information is preferred if present. When not available, we look at the location field in users’ profiles and geocode that location. If neither source of information is available, we check for information in their tweets, but only in cases where the *place_id* field of the API response returns relevant information. As a result, a considerable portion of user locations in our dataset could be identified only to city or postcode level. Upon identifying the location of users, we remove users located outside the United Kingdom or whose location we could not identify up to postcode level. This reduces our dataset to 565,028 messages or 11% of all collected messages; a sample of messages deemed sufficiently large [39] to allow for exploring the geographic dispersion of Vote Leave and Vote Remain Campaigns.

We rely on the use of highly-charged hashtags (i.e., takebackcontrol or lovenotleave) as a proxy to users’ ideological position. For each tweet, we count the number of hashtags advocating the Leave and Remain campaigns. We tag the message as Remainer or Leaver on the basis of the highest number of hashtags used. Messages without hashtags advocating one of the campaigns are tagged as Neutral. This information, once aggregated, additionally serves to identify the affiliation of users that tweeted or retweeted politically polarized hashtags. Highly polarized messages―i.e., tweets including several one-sided hashtags, are however uncommon. For users championing the Vote Leave campaign, only 16% of their messages included more than a single one-sided hashtag. These messages are yet more uncommon in the Remain campaign, where only 2% of messages included more than one hashtag clearly associated with that side of the campaign.

We subsequently identify the campaign affiliation (Leave or Remain) of users @-mentioned or retweeted in the original tweet. To achieve this, we loop through the dataset to find messages tweeted by these recipients that championed either side of the campaign. We calculate the mode or “mean affiliation” per user based on the frequency of one-sided hashtags used throughout the period. The mean affiliation per user can only be calculated for users that actively participated in the referendum campaign on Twitter. In other words, for users at the receiving end (@-mentioned or retweeted) to be identified as Leaver or Remainer, the user in question must have tweeted or retweeted a separate tweet with hashtags clearly aligned with one side of the campaign, whereas users that tweeted an equal number of Remain and Leave hashtags are tagged as neutral. The rationale for restricting the parameters of ideological identification between users was to avoid mainstream media and high-profile accounts, which are regularly @-mentioned or whose tweets are retweeted with the addition of one-sided hashtags, to be classified in either side of the campaign battle. The mean affiliation has the added benefit of filtering out retweets or @-mentions intended as provocation or ironic remark; these messages are offset by the broader ideological orientation tweeted by the account, and users that have only sourced information or received @-mentions are classified as neutral for not having themselves tweeted any partisan hashtag.

In short, we opted for a more conservative approach to identifying campaign affiliation at the receiving end of a tweet so that users are only associated with one side of the campaign if the user herself tweeted a partisan message at some point during the campaign. We believe this approach grounded on the mean affiliation per user reflects strong campaign membership with low probability of false-positives. These conservative parameters to identifying campaign affiliation further reduced our dataset to 33,889 tweets, the unit of analysis used in this study, posted by 15,299 unique users. Ultimately, the multiple sampling of the data (timespan, geographic location, campaign affiliation of sender and receiver) rendered a highly curated dataset comprising ideological markers and geographically enriched data. Given the rationale of this project, we believe this dataset offers a defensible if limited representation of the debate and our conclusions are conditional on these constraints.

We followed the directionality of the information to graph a network of @-mentions and retweets, with *A*→*B* when *B* retweets *A* and *A*→*B* when *A* mentions *B*. We operationalize echo chambers as a function of the identified campaign affiliation. We tag each tweet as in-bubble if sender and receiver (@-mentioned or retweeted) have tweeted the same campaign. We tag the tweet as cross-bubble if the sender has tweeted one campaign and the receiver (@-mentioned or retweeted) has tweeted the opposite campaign. We tag the tweet as out-bubble if either sender or receiver was classified as neutral, which means any of them have not tweeted messages with clearly supportive campaign hashtags. Lastly, we deployed a bot detection protocol [40] that led to the identification of 237 users with suspicious bot activity whose echo chamber activity was limited to only 63 messages. To control for potential issues associated with bot activity, we replicated the analysis without this group of users, but the test did not yield significantly different results.

With the location of users defined using the abovementioned triangulation approach, we leverage the longitude and latitude values to calculate the Euclidean distance (in kilometers) covered by the sender and receiver of @-messages and retweets. We use the canonical mean equatorial radius (6378.145 km or 2.092567257E7 ft.) for earth radius. As such, our calculation is not mathematically precise due to the inaccurate estimate of the earth’s radius (R). Despite this perennial limitation, we believe the calculation is adequate as mathematical precision is of lesser importance when analyzing data whose geographic accuracy is limited to postcode level. We repeat the process for each tweet, thus identifying the account being @-mentioned or retweeted and calculating the distance (in kilometers) between sender and receiver.

Finally, differences in distance are analyzed with a series of statistical tests, including linear regression, Chi-square, and Kolmogorov–Smirnov. For the Chi-squared tests, we reject the null hypothesis of the independence assumption if the *p*-value of $x^{2}=\sum_{i,j}\frac{{\left(f_{i,j}–e_{i,j}\right)}^{2}}{e_{i,j}}$ is less than the given significance level *α*. To test Hypotheses H5, we examine if the variables sender’s affiliation and receivers’ affiliation are independent and if the probability distribution of one variable is affected by the other.

### Limitations of the method

Identifying the location of social media users is a notoriously difficult task given the multitude of geographic information made available by social media platforms with various levels of accuracy, reliability, and granularity. While only 1% of tweets usually include geolocational information [41, 42], we have maximized this source by relying on the Twitter REST API to collect the 3200 messages available per user and searched for geolocation information in their tweets. Upon identifying a positive match, we apply this location to tweets authored by the same user that lacked geographic information. This approach maximized precision in determining the location of users, but there is no way of knowing whether the geolocation refers to a place where the user works, studies, lives, or was simply traversing. The same ambiguity pervades information made available in the user profiles, which furthermore may be entirely fabricated. In view of these caveats, we do not expect the geocode and profile data to necessarily reflect users’ home or work location. Instead, we rely on this signal as geographic markers between two users sharing political homogenous information. In other words, this study explores the interaction between different geographic locations and ideological affiliations as opposed to surveying the residence of Twitter users in the United Kingdom. Lastly, we relied on the HERE API to geocode and reverse geocode geographic location. As the API provides attribute-level information about the match quality, we leveraged this information to remove API responses with a MatchCity score >.9 and whose field MatchType of pointAddress failed to pinpoint the location on the map [43].

## Results

We evaluated H1 and H2 by testing whether the probability distribution of the sender’s and receiver’s affiliation are independent―i.e., one variable is not affected by the presence of another. The variables are significantly correlated (r = .66, *p* < .0001) with a Chi-squared value of 9646.4 (*p*< 2.2e-16). As a result, we conclude that the two variables are in fact dependent. We further explored H1a and rejected the null hypothesis of the independence assumption at the 95% confidence level, as users tweeting the Leave campaign are significantly more likely to interact with users also tweeting highly partisan Leave hashtags. In fact, only 9% of messages tweeted by users affiliated with the Leave campaign were directed to users associated with the Remain campaign (cross-bubble communication). This contrasts with 22% of interactions directed to neutral users (out-bubble communication) and a towering 69% of Leave @-messages and retweets being sourced from or directed to another user affiliated with the Leave campaign (in-bubble communication), with little to no difference between @-mentions and retweets.

**Fig 1:**
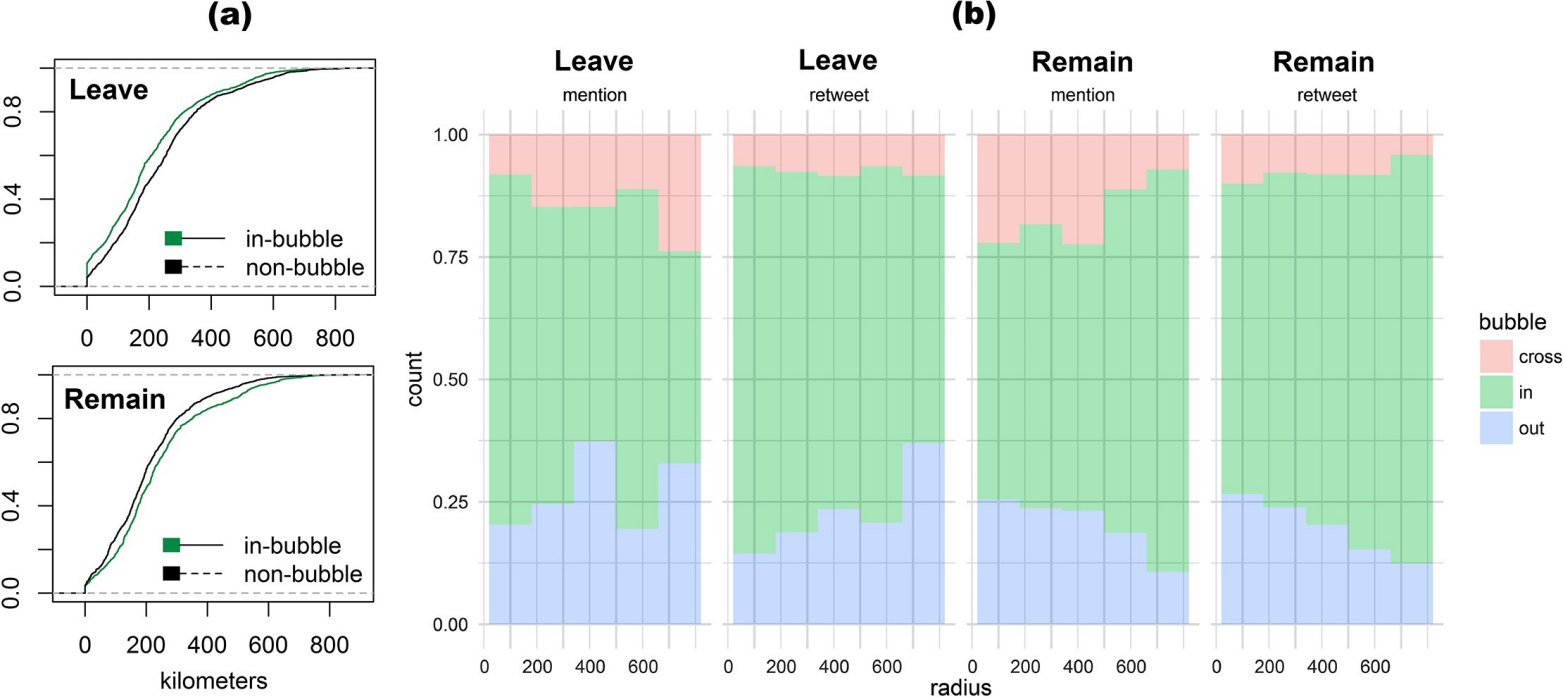
(a) Cumulative Distribution Function (CDF) of in-bubble (echo chambers) and non-bubble (out- and cross-bubble) communication; and (b) Histogram of distance travelled by messages between sender and receiver in 50-kilometer bins.

The intensity of echo-chamber communication is remarkably similar on the Remain side of the campaign (H1b), where only 10% of users directed @-mentions or retweeted content from users identified with the Leave campaign (cross-bubble communication), with 22% of interactions including neutral users (out-bubble communication), and a total of 68% of interactions initiated by Remainers being echo chambers (in-bubble communication). The likelihood of users campaigning for one side of the referendum engaging with users of the same leaning―instead of neutral or adversarial users―was captured by fitting a linear regression model on the sender’s affiliation as the explanatory variable of echo-chamber communication: partisan affiliation explains nearly half of the variance in the data (*R*^*2*^_*adj*_ = .44, *p*<2.2e-16). Fig 1 unpacks these findings and shows the prevailing patterns of echo-chamber communication compared with out-bubble and cross-bubble communication (complementarity), both in the Leave and the Remain campaign, across a range of distance radiuses.

We approached H2 by examining whether Leave and Remain interactions are predominantly within neighboring in-bubbles or geographically proximate echo chambers―i.e., within a 50-kilometer radius expanded in 50-kilometer increments up to 900 kilometers―which is the maximum straight-line distance between two geographical points in the United Kingdom (from Land’s End to John o’ Groats). We found that most interactions are within a 200km radius, but the geographic trend is different between the Leave and Remain campaigns. As shown in Fig 1A, the Cumulative Distribution Function (CDF) of in-bubble Leave messages covers shorter distances compared to non-bubbles (i.e., out- and cross-bubbles), with half of in-bubble messages covering less than 200 kilometers. The trend is reversed on the Remain side of the campaign, in which in-bubble interactions cover longer distances compared to non-bubble messages.

Fig 1B also shows that Leave-campaign messages are chiefly exchanged within ideologically and geographically proximate echo chambers, a component of echo-chamber communication behavior that we further unpack in the following analyses. There is also relatively little cross-ideological retweeting and @-mentioning, much in line with previous findings reported in the literature [35]. While in-bubble is also the prevailing mode of communication on the Remain side of the campaign, the trend is however inverted: as distance between sender and receiver increases, in-bubble communication becomes more common and covers increasingly larger geographic areas compared to out- and cross-bubble interactions. This reversed trend depicted in the CDF plot is also captured by the mean distance covered by Leave messages, at 199km for in-bubble and 234km for non-bubble ($\tilde{x}$ = 168 and $\tilde{x}$ = 208, respectively). For Remain messages, contrariwise, the mean distance is 238km for in-bubble and 204km for non-bubble ($\tilde{x}$ = 209 and $\tilde{x}$ = 184, respectively).

**Fig 2:**
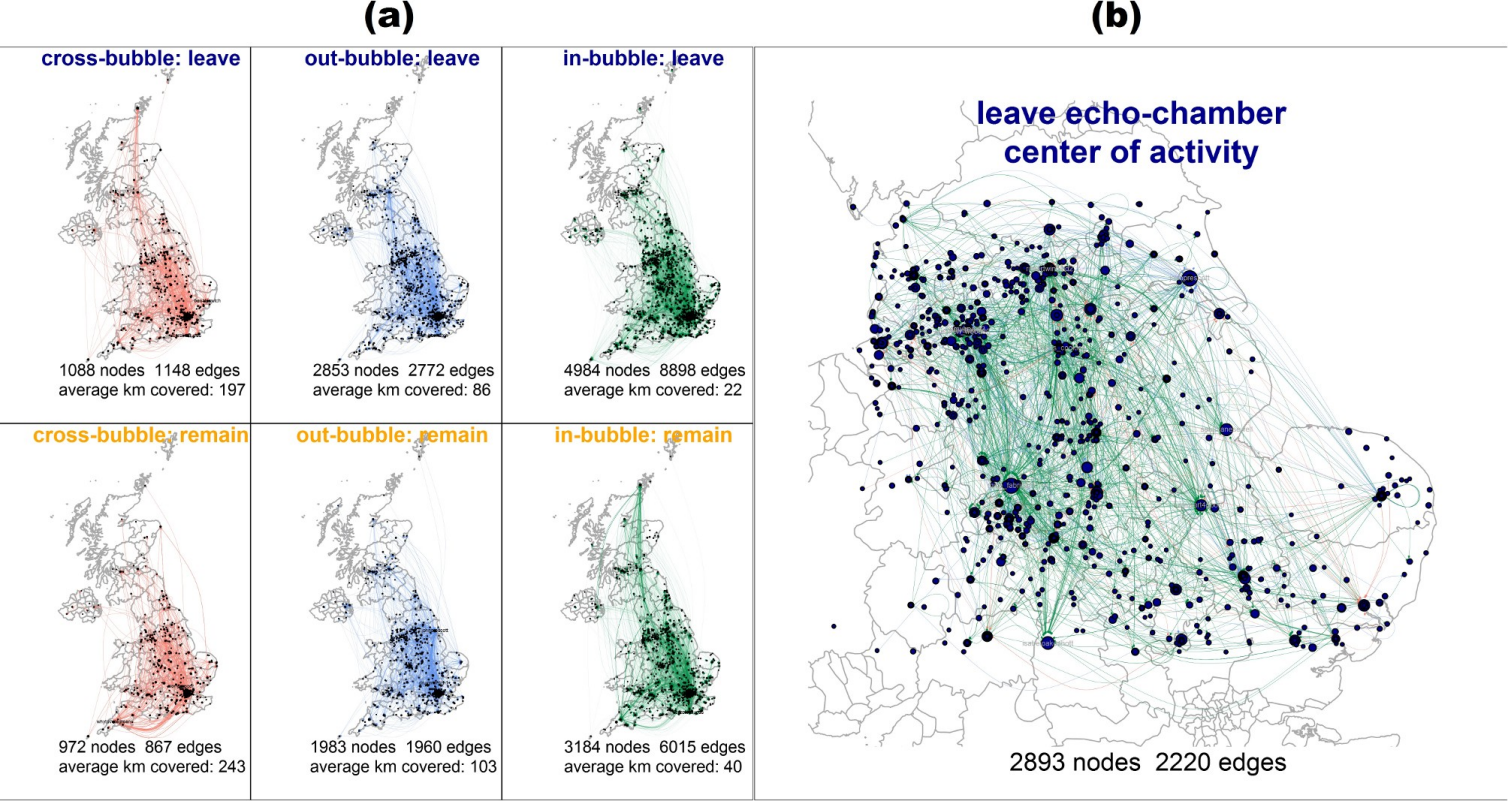
(a) Geographic pattern of cross-bubble, out-bubble, and echo chambers (in-bubble) with number of vertices and edges in each subgraph and the average distance travelled between sender and receiver; and (b) snapshot of the central point of diffusion of the Leave campaign, geographically located in the English Midlands, the North, and the East.

This is consistent with Hypotheses H2a and H2b, which state that Leave and Remain interactions, respectively, are predominantly within neighboring in-bubbles. Fig 2 shows this relationship by contrasting cross-, out-, and in-bubble communication across the United Kingdom (we found no difference in the communication patterns of @-mentions and retweets). The results lend support to H3a but reject hypothesis H3b, as only Leave echo chambers are likely to cover short geographic distances compared with non-bubbles. In fact, there are nearly three times as many in-bubble interactions in the Leave campaign for every cross-bubble and out-bubble communication combined, with the number of users involved in out-bubble communication being about half and one-fifth, respectively, of those involved in echo-chamber communication.

Despite echo chambers on the Remain side being independent from geographic distance, there is a much higher ratio of interactions falling within in-bubble patterns. Similarly to the Leave campaign, there are three times as many in-bubble interactions for every out-bubble, and six times as many for every cross-bubble interaction. The number of Remain supporters involved in out-bubble communication is about two-thirds of those involved in in-bubble communication and only a third if we compare cross-bubble with in-bubble. The quantifiable higher volume of in-bubble communication has potential implications on the homophily patterns observed in cross, out, and in-bubble, as larger groups are likely to be more homophilous compared with smaller randomized subsets of the same group [13, 44]. Yet, while echo chambers in the Leave campaign appear constrained by short geographic distances (H3a), this is not the case on the Remain side (H3b). In fact, Remain echo chambers are likely to span greater geographic distances while their cross-bubble communication is physically concentrated around neighboring communities, an indication that users aligned with the Remain campaign tried to cross the ideological divide within their communities.

We approach H4, which hypothesizes that Leave interactions cover shorter geographic distances compared with Remain interactions, by calculating the average distance @-mentions and retweets travelled from source to destination for each side of the partisan divide. The absolute differences observed across campaigns lend support to H4, as one-quarter of Leave interactions took place within 100 kilometers whereas fewer than one-fifth followed such pattern in the Remain campaign. The average distance covered by Leave partisan messages is also shorter at 178km compared with 199km for the Remain campaign. In-bubble communication in the Leave campaign covers considerably shorter geographic distance of only 22km compared with 40km for the Remain campaign. The pattern persists in out-bubble and cross-bubble communication, where Leave messages cover 86 and 197 kilometers compared with 103 and 243 for Remain messages, respectively. Fig 2 unpacks these differences and shows the geographic clustering of Leave messages, particularly in-bubble interactions, centered in the Brexit heartland of the English Midlands, the North, and the East.

We approach H5a, which hypothesizes that echo-chamber communication is associated with geographic proximity in the Leave and Remain campaigns (H5a and H5b, respectively), by comparing the density distribution curves of in-, out-, and cross-bubble communication subgraphs alongside the density curve of randomly-generated comparable subgraphs. To this end, we randomly swap the location of users in each subgraph (in-, out-, and cross-bubble), recalculate the distance travelled by @-mention and retweet messages, and compare the observed distribution of distances against the random distribution of distances travelled by each message. The rationale for this analysis is to identify distributions that deviate from the random reallocation of users across geographic locations while preserving individual social networks identified by their communications on Twitter as well as the geographic distribution of users in the country. We do not assign random locations to users; we simply swap the location of users in each subgraph to test if the distribution is similar to the random network which preserves the overall geographical distribution of users. This approach establishes an association between echo-chamber communication and the geography of message diffusion whenever the observed networks―*ceteris paribus*―differ significantly from the random network. In other words, for each iteration of the test we retain the set of locations in each subgraph, but randomly reorder the locations to test whether geographic dependencies found in echo-chamber communication are replicated in the randomized geographic network.

**Fig 3:**
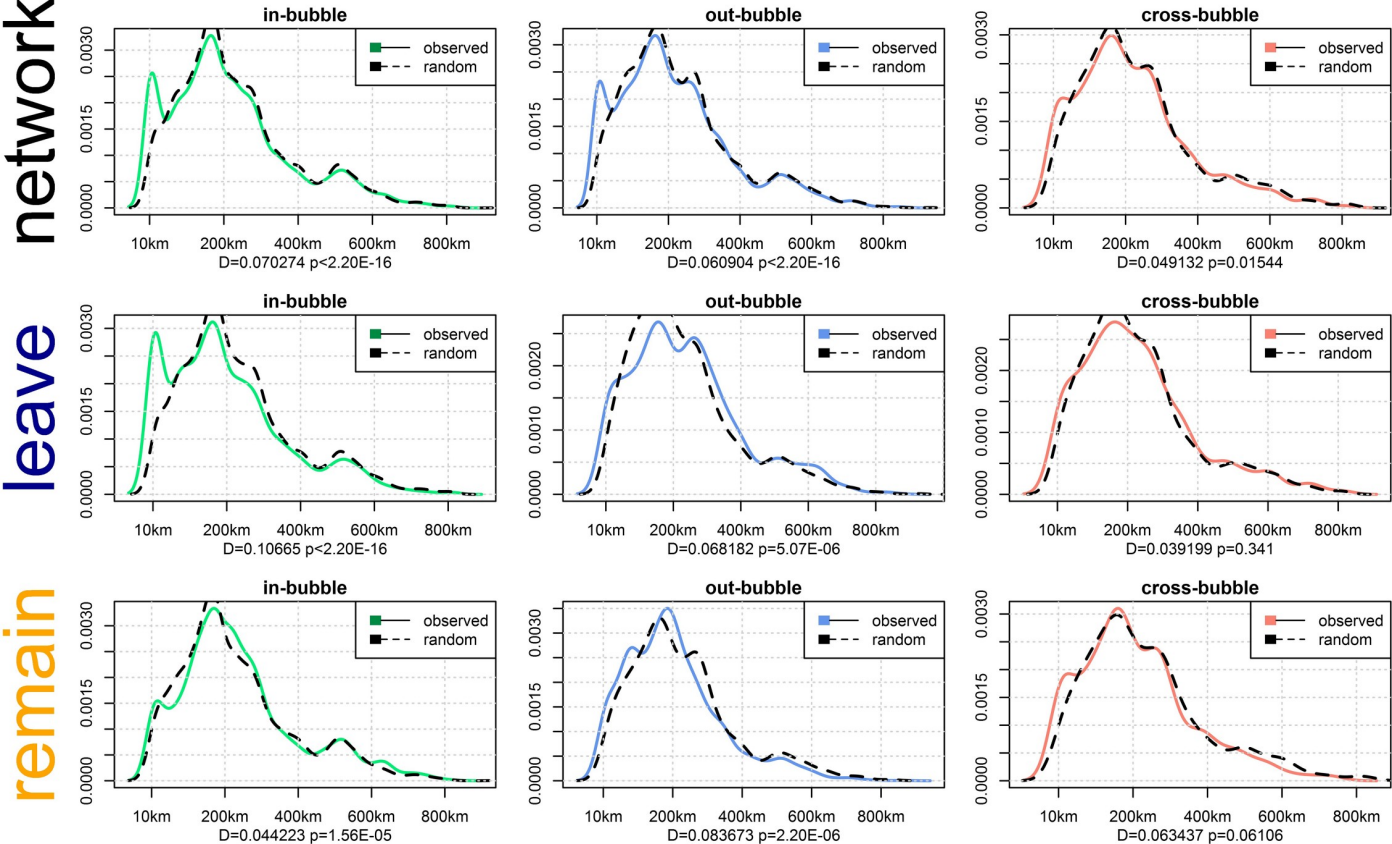
Distances covered by interactions across in-bubble, out-bubble, and cross-bubble for referendum network and subgraphs of the Leave and Remain campaigns, followed by the Kolmogorov–Smirnov test statistic and *p*-value. The dotted line shows the reference probability distribution used to test the similitude of the two samples with a continuous distribution.

We ran 100 iterations of each test and the results are summarized in Fig 3: the high volume of interactions within geographically proximate echo chambers―i.e., within the 50 kilometers radius―is a considerable departure from the distribution in the randomized network. This deviation is particularly prominent in echo-chamber communication (i.e., in-bubble interactions). This pattern disappears when the location of users is randomly reshuffled, an indication that the distribution is not determined by chance. We thus conclude that the geographic distribution of echo-chamber communication is unlikely, i.e., much less likely to happen than in the randomized null model. This unlikely distribution is yet more salient in the subgraph of Leave in-bubble interactions and disappears in out- and cross-bubble interactions for the Leave campaign and again in the entire network of Remain interactions. In other words, the association between geographic proximity and echo-chamber communication is restricted to the Leave campaign and lends support to hypothesis H5a, while in the Remain campaign we observe no such dynamics and hence reject hypothesis H5b.

To assess the significance of these results, we performed a Kolmogorov–Smirnov test on the probability distribution of distances covered by messages compared with the probability distribution of distances covered by messages with users’ locations randomly reshuffled. In other words, we swap the location of users in the graph and calculate the distances covered by their interactions again, thus providing a reference probability distribution to test the similitude of the two samples with a continuous distribution. Fig 3 shows the test statistic, the maximum distance between the Empirical Cumulative Distribution Function (ECDF) of the two samples, and the *p*-value for each of the tests. The results are significant for all modalities of self-selected bubble and each side of the political divide, except for cross-bubble communication, which is not significant in any of the subgraphs, likely a result of the small sample size of cross-bubble communication as the distributions are similar with no superimposed oscillatory disturbances.

The maximum distance (supremum) between the CDFs of the two samples is significantly higher for Leave in-bubble interactions, in which the peak amplitude deviates from the pattern observed for the rest of the network and during the random reshuffling of users’ locations. The probability of seeing a test statistic as high or higher than the one observed if the two samples were drawn from the same distribution is vanishingly small. The results thus support hypothesis H5a: echo-chamber communication in the Leave campaign is likely to be associated with geographic proximity, with in-bubble interactions in the Leave campaign showing significantly more short distance activity than expected by chance (*p*<2.20E-16). Hypothesis H5b―that echo-chamber communication is associated with geographic proximity in the Remain campaign―is also accepted; echo-chamber communication among the Remain camp is associated with geographical distance (*p*<1.56E-05), but the effect is essentially reversed: echo-chamber communication in the Remain campaign is more likely to cover larger distances compared with out- and cross-bubble communication, which on average cover shorter geographic distances.

In view of the high deviation from the probability distribution of users’ locations randomly reshuffled, we sought to further examine hypothesis H5 by probing variables that could have interfered with this distribution. We firstly speculated that highly-active, super-users in a few cities could have drawn the geographic distribution of in-bubble communication in the Leave campaign. Secondly, we conjectured that isolated events such as the murder of the Labour Member of Parliament Jo Cox could have likewise skewed a distribution that would otherwise remain comparable to the remainder of the campaign data. However, we managed to rule out these effects by inspecting the probability distribution while controlling for these variables. To this end, we performed Kolmogorov–Smirnov tests on the observed subgraph of echo-chamber communication in the Leave campaign and the same subgraphs minus super-users (maximum 10 tweets). The results rejected the hypothesis that the two distributions are significantly different at the 95% confidence interval and supports the assumption that the geographic patterning found in echo chambers is independent of super-users.

**Fig 4:**
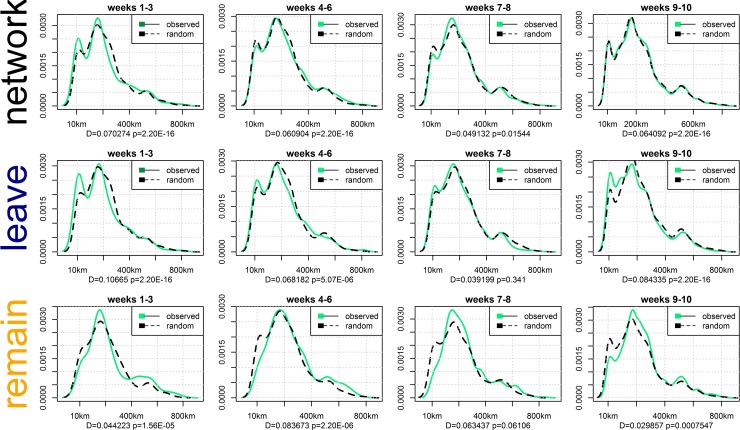
Distribution of distances covered by echo-chamber communication (in-bubble) for the referendum network and subgraphs of the Leave and Remain campaigns over the 10-week referendum campaign (14 April to 23 June). The Kolmogorov–Smirnov test statistic and the *p*-value indicate the differences in the observed and randomized distributions: while in weeks 9–10 observed and randomized signals are similar at the network level, there are remarkable and inversed interactions between online activity and geography in the Leave and Remain subgraphs.

We addressed our second conjecture by slicing the 10-week period covered by the referendum campaign (14 April to 23 June) into four sub-periods comprising weeks 1–3, weeks 4–6, weeks 7–8, and weeks 9–10 and performing Kolmogorov–Smirnov tests on each temporal scenario. The distribution appears to change over time, but the geographic patterning associated with echo chambers in the Leave campaign remains relatively robust throughout the period. Fig 4 shows the observed and random distribution for echo chambers in the referendum network and subgraphs of the Leave and Remain campaigns over the 10-week period. Weeks 1–3 show a similar peak to the one observed in the aggregate network, which decreases in the weeks 4–8 but surges again in the last two weeks of the referendum, where most of the user activity is concentrated. It is interesting to note that in weeks 9–10, which are marked by the intense activity of Leave and Remain campaigners, the observed distribution is patently similar to the randomized signal at network level, but the separate inspection of Leave and Remain subgraphs reveals striking interactions between online activity and geography.

In summary, the weekly variations continued to present peak amplitudes that deviate from the rest of the network and from the distribution observed with the random reshuffling of users’ locations. The tests thus reveal an inverse geographic patterning of echo chambers for Leave and Remain campaigns, particularly in the weeks leading up to the vote. Therefore, we conclude that echo chambers in the Leave campaign are significantly associated with geographic propinquity and the results appear to be robust across classes of Twitter users and during different moments of the referendum campaign.

## Discussion and conclusion

This study identified and substantiated the existence of geographically-bound sociopolitical enclaves materializing in polarized echo-chamber communication online. The first hypothesis tested in this study is broadly consistent with previous results found in the literature [1, 2] and research that reported significant evidence of echo chamber behavior in the Brexit debate on Facebook [9]. The finding that Remainers were more likely to engage in cross-bubble communication is also consistent with previous research that found liberals more likely to engage in cross-ideological retweeting than conservatives [45]. The results of hypothesis H1 are thus broadly consistent with previous research: the Brexit debate accentuated the political divides among the British public along antagonistic fault lines. As such, it is unsurprising to have found Leave and Remain campaigns engaging in widespread echo chamber behavior.

It is the results of hypotheses H2-H5 that shed new light on the dynamics of echo-chamber communication. By identifying a geographic patterning in online echo chambers, particularly in the Leave campaign, where in-bubble communication was restricted to physical communities proximate to each other, we have pinpointed one force driving echo chambers that is exogenous to online interaction. We nonetheless expect others to be at play, with the geographic patterning observed hitherto likely interacting with sociodemographic variables that reportedly marked much of the Brexit debate [46, 47]. While these findings advance the study of echo chambers by revealing how geographic distance interacts with this segregative pattern of communication, further research is necessary to examine the claim that politically homogeneous communication is associated with geographic distance.

Hypothesis H2-4 also work as a control mechanism. We previously established that echo-chamber communication was prevalent in the Brexit debate, and that in-bubble interactions were more likely to cover short geographic distances for the Leave campaign even after controlling for highly-active users and seasonal variations. Yet, only by inspecting the entire network, along with out- and cross-bubble subgraphs, one could identify the geographic dependency as a development restricted to echo-chamber communication. In other words, by extricating in-, out-, and cross-bubble communication we managed to differentiate the extent to which geographic propinquity relates to being exposed to consonant information versus being exposed to heterogeneous information.

Our results call into question the assumption that echo chambers are a communication effect resulting from online discussion alone. The analysis of the data sought to identify the extent to which geography was endogenous to the echo-chamber subnetwork. The null hypothesis was that the unequal geographic distribution of the British population was to be observed at each iteration of the tests, with Greater London remaining the central point of information diffusion and echo-chambers reappearing with relatively unchanged geographic coverage. While the network topology remained the same at each iteration, along with the distribution of users’ location, the geographic distribution of echo chambers was significantly different. The bell-shaped, near-normal distribution of in-, out-, and cross-bubbles in the randomized networks present a significant departure from the observed geographic coverage of echo chambers and suggests that geography is an exogenous force impinging on actors involved in echo-chamber communication during the Brexit debate.

The absence of geographic factors interacting with echo chambers in the randomized networks is puzzling because it deviates both from the geographic propinquity of echo chambers observed in the Leave campaign and the geographic remoteness observed in the Remain camp. The results thus suggest that the geographic proximity of Leave echo chambers, and the geographic remoteness of Remain echo chambers, are likely driven by the physical clustering of fundamentally disparate social networks. Instead of incorporating remote strangers that are activated and incorporated as organic members of one’s social network [48], the results suggest a spill-over from in-person interaction patterns to online social networking sites, a causal hypothesis about the direction of homophily that our data cannot test definitively. In other words, and despite the anxieties triggered by filter bubble and echo chamber in social platforms, the results suggest that echo chambers are connected with homophilous dependencies in physical social networks that are not as such created social media activity.

The results are particularly interesting when considering that the geographic propinquity of online echo chambers was restricted to the Leave campaign. In sharp contrast, Remainers appear to have focused their cross-campaign efforts on neighboring areas of their community, whereas their echo-chamber communication extended to more distant users. This is nonetheless in line with the hypothesis that physical social networks are likely to have spilled-over to online debate. The fundamental differences in echo-chambers in the Leave and Remain campaigns correspond to the demographic makeup of their social networks, with the latter bringing together individuals who live, work, and study in locations different and often distant from their hometowns [47]. We thus estimate that the significant geographic variation found in the data was likely driven not only by the locations where the two camps were respectively concentrated in, but also the social positions underpinning the geographical location or their constituencies.

The different social positions occupied by Leavers and Remainers is consist with the geographical splintering of the country expressed in the referendum and mirrors the antagonism separating urban loci of political and economic power epitomized by London and the economically fragile northern Britain. Survey literature has long recognized the relationship between spatial clustering and nonresponse in household surveys [49]. Contact probability decreases in metropolitan areas where cultural events are more likely to happen, with city-dwellers spending more time shopping or exploring entertainment options outside of their neighborhoods, thereby leading to lower first contact success rates in large metropolitan statistical areas [50]. With cities being hubs of the national and global economy, it is unsurprising that individuals living in urban areas would travel more and that their resulting social networks be more widely connected. The communication distances covered by their interactions should therefore also be lengthier compared with those inhabiting rural or low-density areas of the country.

Further studies should seek to establish the magnitude of the effect and the underlying mechanisms through which physical proximity and political affinity translates into in-bubble, echo-chamber communication. We expect more intricate relationships between existing physical ties and online interactions to be at play [38, 39], with the relationship between campaign affiliation and geographical propinquity capturing only secondary effects of this interaction. Ultimately, the results reported in this study challenge the assumption that echo chambers emerge from online interactions by uncovering a relationship between extant physical ties and echo-chamber communication. If anything, the results suggest that the collapsing of distances brought by internet technologies may foreground the role of geography within one’s social network.

## Supporting information

S1 File The search criteria used to query the API. (TXT)
